# The brain entropy dynamics in resting state

**DOI:** 10.3389/fnins.2024.1352409

**Published:** 2024-03-26

**Authors:** Xiaoyang Xin, Jiaqian Yu, Xiaoqing Gao

**Affiliations:** ^1^Center for Psychological Sciences, Zhejiang University, Hangzhou, China; ^2^Preschool College, Luoyang Normal University, Luoyang, China

**Keywords:** dynamics, brain entropy, resting-state fMRI, general cognitive ability, human connectome project

## Abstract

As a novel measure for irregularity and complexity of the spontaneous fluctuations of brain activities, brain entropy (BEN) has attracted much attention in resting-state functional magnetic resonance imaging (rs-fMRI) studies during the last decade. Previous studies have shown its associations with cognitive and mental functions. While most previous research assumes BEN is approximately stationary during scan sessions, the brain, even at its resting state, is a highly dynamic system. Such dynamics could be characterized by a series of reoccurring whole-brain patterns related to cognitive and mental processes. The present study aims to explore the time-varying feature of BEN and its potential links with general cognitive ability. We adopted a sliding window approach to derive the dynamical brain entropy (dBEN) of the whole-brain functional networks from the HCP (Human Connectome Project) rs-fMRI dataset that includes 812 young healthy adults. The dBEN was further clustered into 4 reoccurring BEN states by the k-means clustering method. The fraction window (FW) and mean dwell time (MDT) of one BEN state, characterized by the extremely low overall BEN, were found to be negatively correlated with general cognitive abilities (i.e., cognitive flexibility, inhibitory control, and processing speed). Another BEN state, characterized by intermediate overall BEN and low within-state BEN located in DMN, ECN, and part of SAN, its FW, and MDT were positively correlated with the above cognitive abilities. The results of our study advance our understanding of the underlying mechanism of BEN dynamics and provide a potential framework for future investigations in clinical populations.

## 1 Introduction

Spontaneous fluctuations of brain activity are a fundamental feature of the human brain, which can be characterized by blood oxygenation level dependent (BOLD) signal using resting-state functional magnetic resonance imaging (rs-fMRI). The characteristics of these fluctuations are essential to understand the individual differences in brain function and the pathologies associated with neuropsychiatric conditions (e.g., Yu-Feng et al., 2007; Fornito and Bullmore, 2010; Takahashi, 2013). Brain entropy (BEN) is a measure of the irregularity (Wang et al., 2014; Xue et al., 2019; Lin et al., 2022) and complexity (Sokunbi et al., 2013; Wang et al., 2014; Nezafati et al., 2020) of such fluctuations. As BEN can serve as an approximate estimation of neural complexity (Yang and Tsai, 2013; Keshmiri, 2020; Cieri et al., 2021) which can be altered in many disease states, numerous rs-fMRI studies have found BEN can serve as a potential biomarker for various mental or neuropsychiatric disorders (e.g., Yang et al., 2015; Hager et al., 2017; Xin et al., 2022). Two recent studies hypothesized that BEN can also serve as a negative quantification of temporal neuronal coherence which is highly related to general cognitive functions such as memory, attention, and perception (Wang, 2021; Lin et al., 2022). The hypothesis has been supported by negative correlations between BEN and several general cognitive abilities (working memory and fluid intelligence) in the default mode network (DMN) and the executive control network (ECN). It should be noted that such results are in disagreement with previous BEN research: reduced BEN is widely considered to be related to decreased neural complexity which is harmful to cognitive functions (Yang and Tsai, 2013; Keshmiri, 2020; Xin et al., 2021). Therefore, lower BEN is considered to be associated with lower cognitive performance instead of higher cognitive performance.

Despite progress made in this research area, most of the research^1^ treated BEN as temporally stationary throughout the measurement period. However, the resting brain is a highly dynamic system with non-stationary neural activity and rapidly changing neural interactions (e.g., Deco et al., 2011; Preti et al., 2017). Such dynamic information is not likely to be captured by the static BEN analytic approach. A series of studies have demonstrated that the time-varying features of the resting brain can be described by specific reoccurring whole-brain patterns, regarded as “brain states” (Liu and Duyn, 2013; Allen et al., 2014; Fu et al., 2018). The dynamic properties of these brain states are highly related to cognitive and mental processes. Many researchers in the field of dFC (dynamical functional connectivity) have identified FC states from the time-varying resting-state FC matrices. These FC states have clinical relevance with various mental and neuropsychiatric disorders (Kim et al., 2017; Bai et al., 2021; Luo et al., 2021) and are related to cognitive abilities (Li et al., 2017; Nomi et al., 2017; Cohen, 2018). In contrast to the dynamics of FC which represents the synchrony of these brain activities between regions, the time-varying patterns of the brain activity itself are less investigated. Spontaneous fluctuations of BOLD signal are supposed to be generated from mental processes (Raichle and Snyder, 2007), for which they might also have reoccurring patterns related to cognitive processes. One recent study (Fu et al., 2018) investigated the time-varying patterns of ALFF (amplitude of low-frequency fluctuation), one widely used measure for spontaneous fluctuations of BOLD signal itself (reflects regional brain activity strength; Yu-Feng et al., 2007). They identified several ALFF states and revealed that the alterations in the dynamical properties of these ALFF states were associated with disrupted cognitive functions in mental disorders. As BEN is independent of a specific frequency band, it may provide a more comprehensive picture of brain activity than ALFF (Song et al., 2019). Therefore, we hypothesized that BEN, another measure of brain activity itself (i) has time-varying and reoccurring patterns in resting state; (ii) the dynamics of these reoccurring patterns are linked to cognitive functions. Furthermore, since dynamical analytical methods can provide more insights into fundamental properties of brain activity that are not captured by conventional static analytical methods (Hutchison et al., 2013), we hypothesized that our dBEN (dynamical brain entropy) approach, especially by exploring the underlying relations between BEN dynamics and cognitive functions, may help to disentangle the controversial association between “static” BEN and general cognitive ability mentioned above.

In order to verify these hypothesis, we use a large set of rs-fMRI data, aiming to (i) derive dBEN in resting state and identify the reoccurring BEN states using clustering analysis, (ii) explore the associations between temporal properties of BEN states and general cognitive ability.

## 2 Materials and methods

### 2.1 Data acquisition and preprocessing

The rs-fMRI data used in our study are part of the Human Connectome Project (HCP) 1,200 subject release.^2^ The rs-fMRI data are collected from a 3 T Siemens scanner using a standard 32-channel head coil and a body transmission coil. For each participant, four resting scans were acquired with the acquisition parameters as follows: repetition time (TR) = 720 ms, echo time (TE) = 33.1 ms, resolution = 2 × 2 × 2 mm^3^, time points = 1,200. Our study uses the preprocessed HCP dataset labeled as PTN (Parcellation + Timeseries + Netmats) consisting of 812 healthy participants (ages 22–35 years old, 410 females), which also had been used in recent dFC studies (e.g., Nomi et al., 2017; Zhou et al., 2020). The main preprocessing steps for PTN dataset were as follows: rs-fMRI data were firstly processed according to the HCP minimal preprocessing pipeline (Glasser et al., 2013; Smith et al., 2013) using FreeSurfer, which includes B0 unwarping and normalization to MNI-152 template; Second pipeline, HCP netmats pipeline was then adopted to remove artifacts using FMRIB’s ICA-based X-noisifier (ICA + FIX; Griffanti et al., 2014); Group-ICA was performed using FSL’s MELODIC tool to generate group-ICA spatial maps and subject-specific node time series in PTN dataset (Smith et al., 2015).

### 2.2 The framework of dBEN analysis

**Figure 1:**
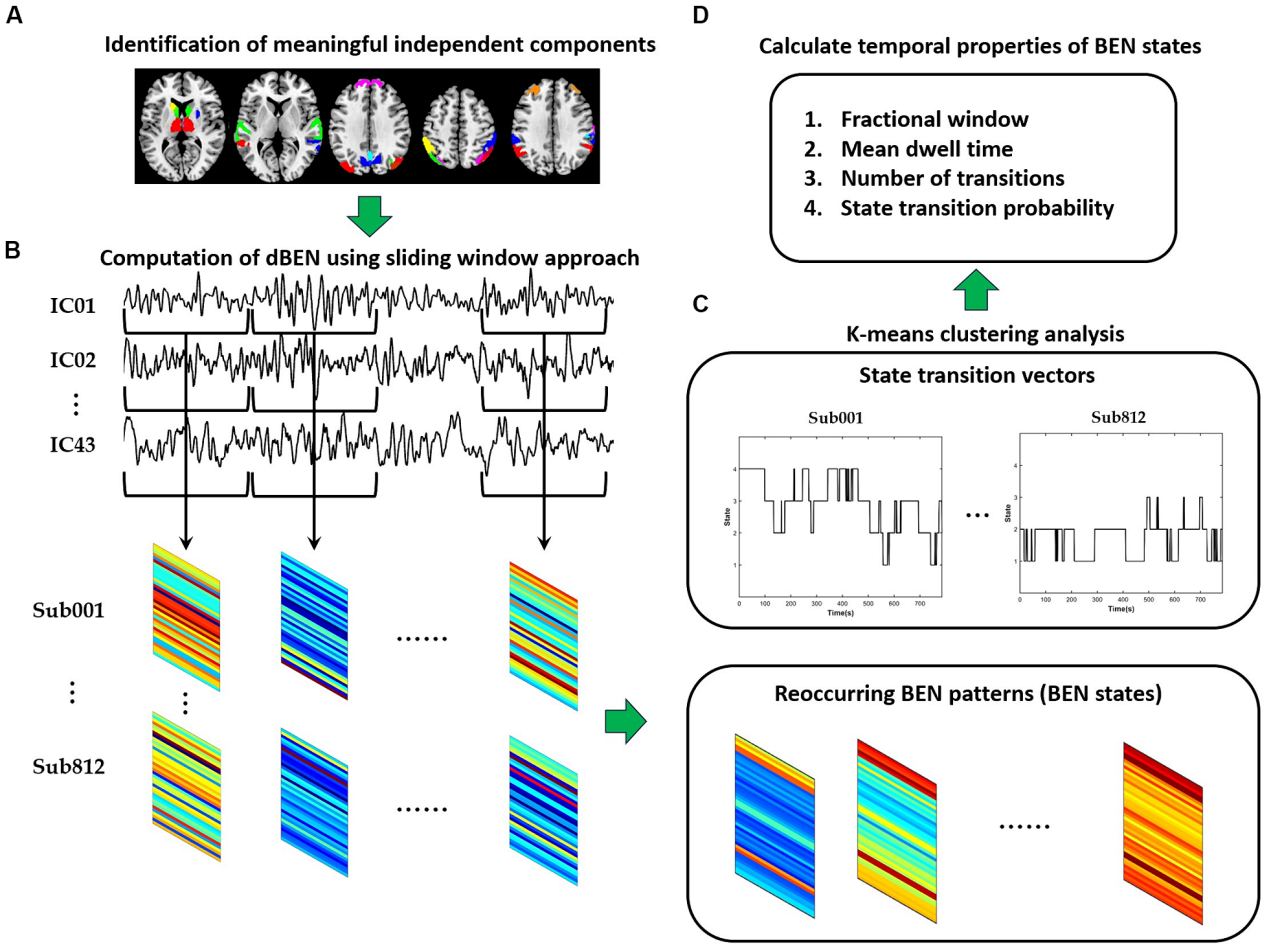
The flowchart of dBEN analysis in the present study. **(A)** Identification of meaningful independent components. **(B)** Computation of dBEN. **(C)** Clustering analysis. **(D)** Calculation of temporal properties of BEN states.

The framework of dBEN analysis is illustrated in Figure 1. Meaningful independent components were first identified according to spatial maps of components (see Section 2.3 for detailed information). A sliding window approach was then adopted to derive dBEN from the time series of the meaningful ICs (see Section 2.4 for detailed information). Several BEN states and their associated dynamics were further derived from dBEN using the k-means clustering method (see Section 2.5 for detailed information). The correlations between temporal properties of BEN states and general cognitive ability were calculated and the significance of each correlation coefficient was tested (see Section 2.6 for detailed information).

### 2.3 Identification of meaningful independent components

**Figure 2:**
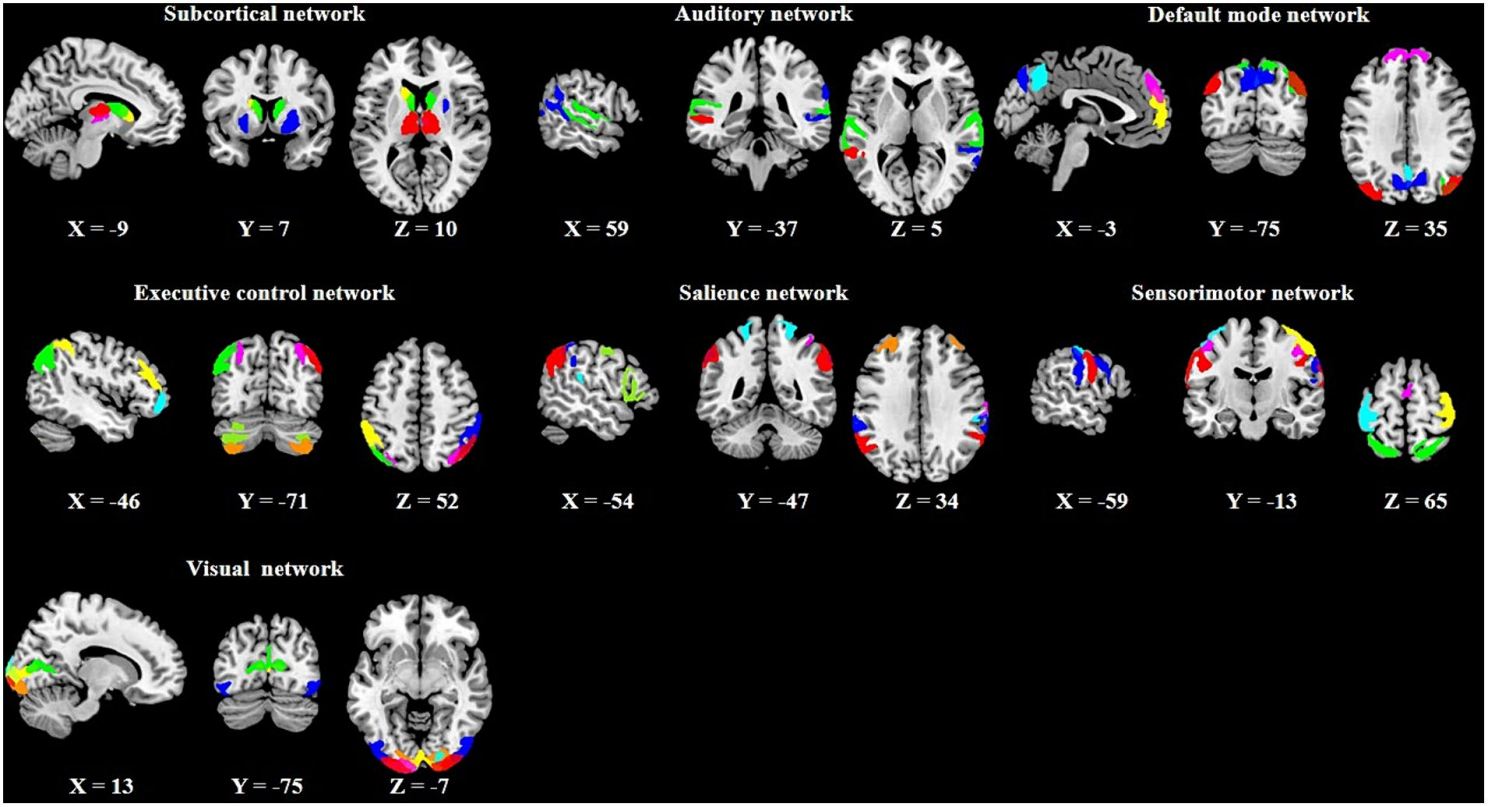
Spatial maps of the 43 meaningful independent components (ICs) and their corresponding seven functional networks.

In the PTN dataset, several different numbers of independent component (IC) parcellations (15, 25, 50, 100, 200, and 300 ICs) were obtained by GICA. We chose the 100 ICs and the first 1,200 time points of these ICs for our analysis according to previous dFC studies (Nomi et al., 2017; Zhou et al., 2020). Out of the 100 ICs, 43 ICs were identified as meaningful components based on the following criteria (Allen et al., 2014): peak coordinates of spatial maps located in gray matter, with minimal spatial overlap with white matter, vessels, ventricles, or susceptibility artifacts, and time courses characterized by a high dynamic range. Based on the Stanford functional ROI template,^3^ we adopted a spatial correlation method to sort the 43 ICs into seven functional networks: subcortical (SCN), auditory (AN), default mode (DMN), executive control (ECN), salience (SAN), sensorimotor (SMN), and visual (VN) as displayed in Figure 2. The detailed information (brain areas, peak coordinates, and correlation coefficients with the template) of each meaningful IC was provided in Supplementary Table S1. The time series of the 43 ICs underwent the subsequent preprocessing steps to reduce physiological and scanner noise: removing the first 10 time points, detrending (regressing linear, quadratic, and cubic trends), despiking using 3D-DESPIKE, and low-pass filtering with frequency cut-off of 0.15 Hz.

### 2.4 Computation of dBEN

The dBEN of the 43 ICs were computed by using a sliding window approach, which is a common computation method for dFC and dALFF. The window type was set to the rectangular window instead of the tapered window used in dFC and dALFF to better capture the features of brain activity itself as suggested by previous studies (Leonardi and Van De Ville, 2015; Mokhtari et al., 2019). According to Yang et al. (2018), data length of at least 97 time points is recommended for the sufficient reliability of BEN analysis, therefore the window length was set to 100 TR in the current study. BEN was calculated by using sample entropy (SampEn), which is a measure of regularity of a time series. Mathematically, SampEn computes the “logarithmic likelihood” that a small section (within a pattern length *m*) of the time series that matches with other sections will still match the others if the section length increases by 1 (Shi et al., 2020). “Match” is defined by a tolerance factor *r*. In our study, the pattern length (*m*) and tolerance factor (*r*) of BEN were set to be 2 and 0.55, respectively, according to the BEN parameter selection method proposed in a previous study (Yang et al., 2018; see more in Supplementary material). The window was slid along time in steps of 1 TR which provides 720 ms temporal resolution for dBEN, resulting in 1191 windows across the entire scan. For each subject, a 43 (number of ICs) × 1,191 (number of windows) dBEN matrix was derived representing the time-varying changes of BEN along with the sliding windows.

### 2.5 Clustering analysis

**Figure 3:**
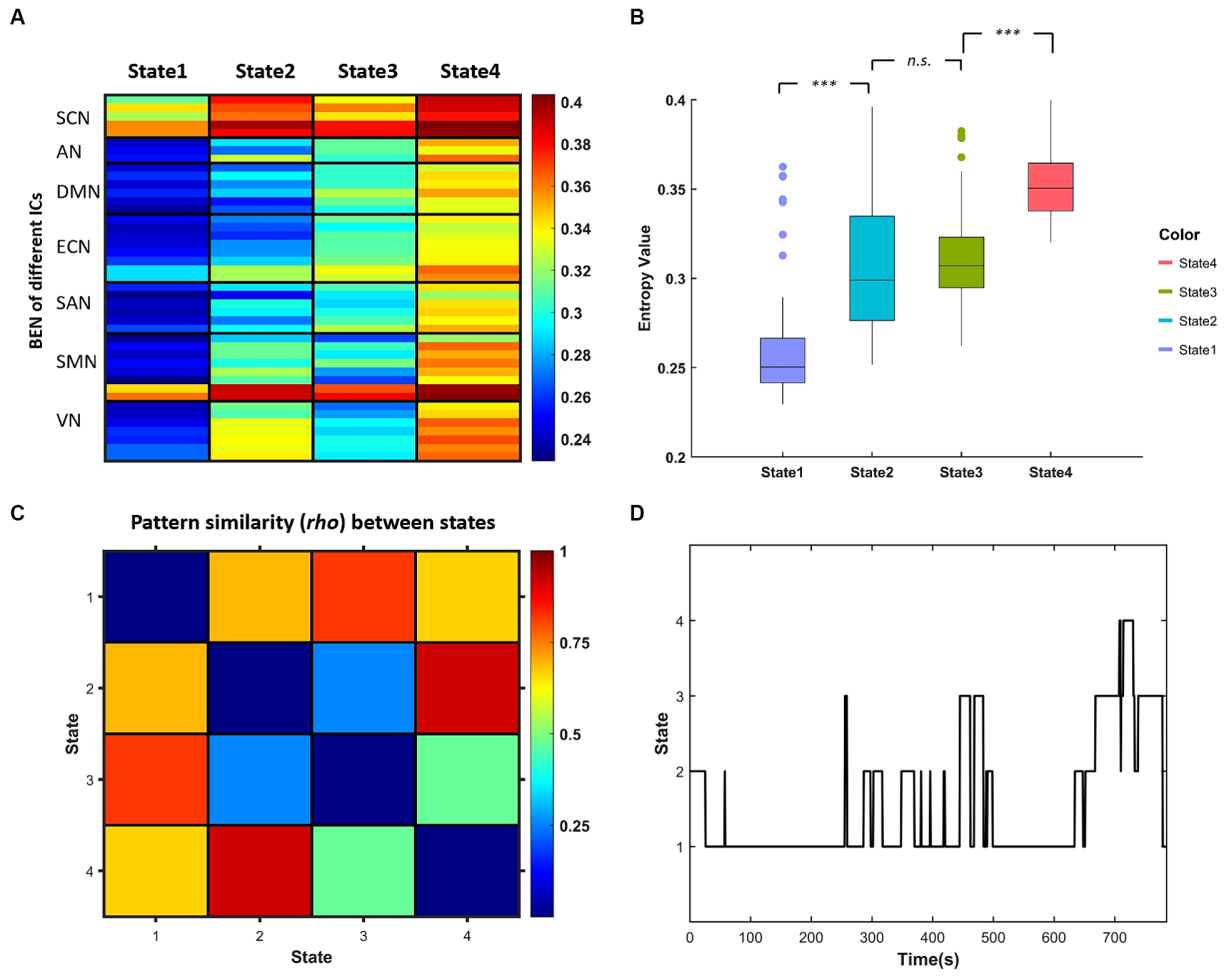
**(A)** Centroids of each BEN states derived by k-means clustering representing in each column. **(B)** Boxplot of centroidal BEN in different state [^***^represents *pcorr* (The pcorr is the corrected *p*-value by FDR method.) <0.001, paired-sample *t* test]. **(C)** Similarity matrix of the centroidal BEN pattern between different states. **(D)** State transition vector of one example subject.

The k-means clustering method was applied to the dBEN matrices of all subjects to estimate the reoccurring dBEN patterns (i.e., BEN States) and their dynamics. The settings of this method were in accordance with previous dFC and dALFF research (Allen et al., 2014; Kim et al., 2017; Fu et al., 2018): L1 (Manhattan) distance was used as the similarity measure of cluster centroids; the optimal cluster number was estimated to be 4 by the cluster number validity analysis (using the elbow criterion, Supplementary Figure S2). The dBEN matrices of all subjects were then categorized into 4 clusters (i.e., States). Thus (i) four cluster centroids of the whole sample and (ii) state transition vectors of all subjects across entire time windows, are derived (Figure 3D).

The state transition vectors were further used to derive the following temporal properties of BEN states: fractional window (FW), mean dwell time (MDT), number of transitions (NT), and state transition probability (STP). The fractional window, measured by percentage, is the proportion of time spent in each state. The mean dwell time is the average duration of time intervals spent in each state, calculated by averaging the number of consecutive windows belonging to one state before changing to another state. The number of transitions is the number of states changed from one to another throughout the entire 1,191 windows. The state transition probability represents the probability of changing from one state to another.

### 2.6 Associations between BEN dynamics and general cognitive ability

General cognitive performance data of the 812 subjects in PTN dataset were also obtained from HCP website. Table 1 provides a summary of these cognitive tests. Detailed information on these tests and references can be found in the HCP Data Dictionary 1,200 Subject Release.^4^ The Pearson correlation (controlling for the effects of age and gender) was adopted to explore the associations between temporal properties of BEN states and cognitive test scores described in Table 1. FDR (False Discovery Rate) was then adopted to correct the *p*-values of the correlations that involve multiple states (i.e., the correlations involving FW and MDT).

**Table 1:** Measures of the cognitive ability in our study.

Cognitive ability	Test name	Outcomes measurement
Episodic memory	Picture sequence memory test	Score based on correct adjacent pictures
Cognitive flexibility	Dimensional change card sort	Combination of accuracy and reaction time
Inhibitory control	Flanker task	Combination of accuracy and reaction time
Fluid intelligence	Penn progressive matrices	Number of correct responses
Processing speed	Pattern completion processing speed	Number of items correct in 90-s period
Spatial orientation	Variable short Penn line orientation test	Number of correct responses
Sustained attention	Short Penn continuous performance test	Median RT of true positive responses
Verbal episodic memory	Penn word memory test	Number of correct responses
Working memory	List sorting	Number of correct responses

## 3 Results

### 3.1 The clustered BEN states

Figure 3A displays the centroids of the four BEN states derived by k-means clustering, and the boxplots of these centroidal BEN are presented in Figure 3B. It can be observed from Figure 3B that: (a) State 1 and State 4 were of the lowest and highest overall BEN respectively, while State 2 and State 3 were of intermediate overall BEN; (b) significant differences in the overall BEN occurred between different pairs of states except between State 2 and State 3. Figure 3C displays the similarity matrix of BEN pattern between different states: (a) high BEN pattern similarity existed between State 1 and State 3, and between State 2 and State 4; (b) the lowest BEN pattern similarity existed between State 2 and State 3. Combined with Figure 3A we can find that: (a) high within-state BEN were all located in SCN and cerebellum (the last two ICs of SMN) in different states; (b) the location of low within-state BEN differed between different states and such difference was presented between State 2 and State 3—low within-state BEN in State 2 were mainly located in DMN, ECN and part of SAN while those in State 3 were mainly located in SMN and VN.

### 3.2 Temporal properties of BEN states and their associations with general cognitive abilities

**Figure 4:**
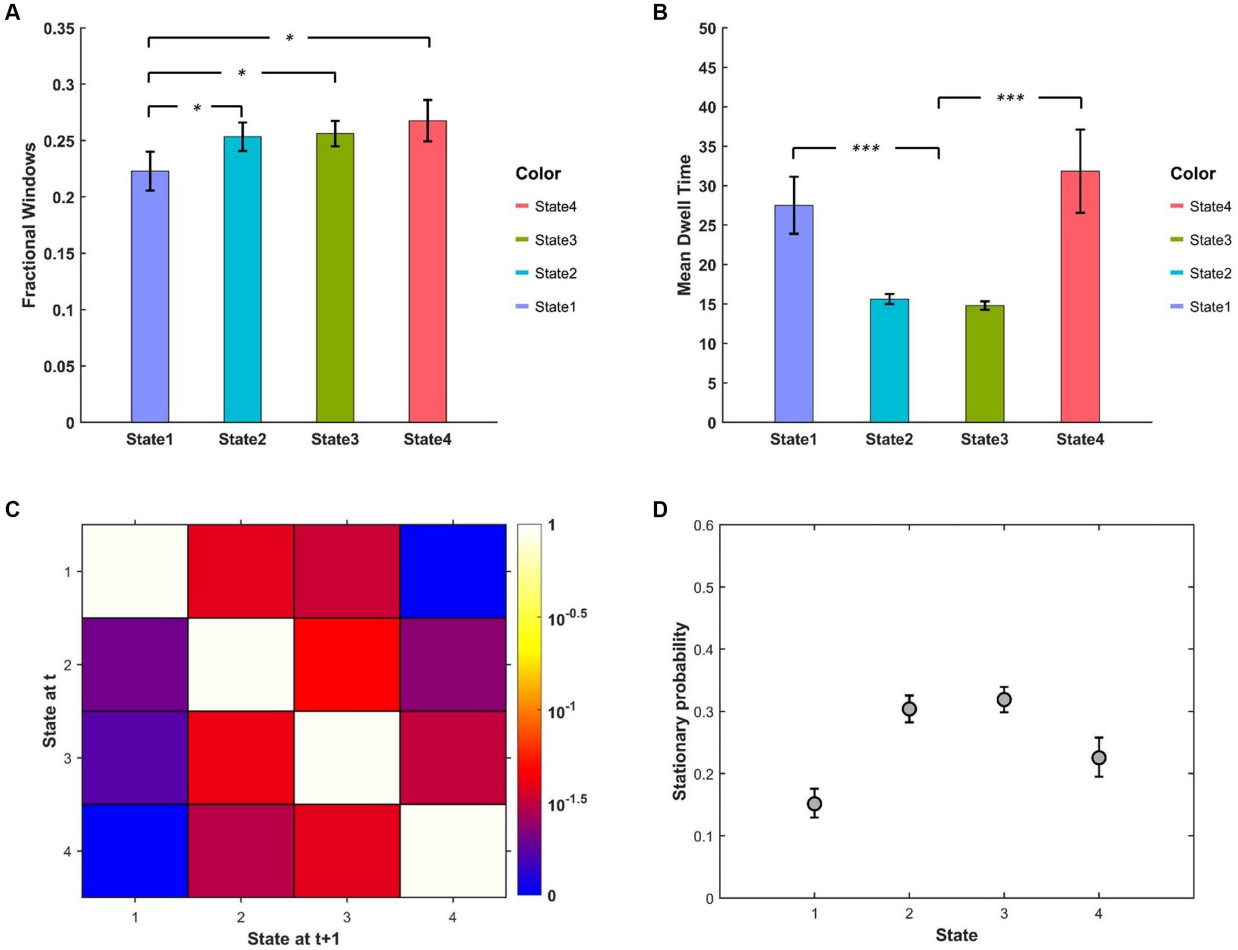
**(A)** FW and **(B)** MDT between different BEN states. **(C)** State transition matrix averaged over subjects, the element in *i*th row and *j*th column represents the transition probability from State *i* to State *j*. **(D)** Stationary probabilities of different BEN states, error bars represent the 95% confidence intervals obtained from 3,000 bootstrap resamples of the stationary probability matrix.

The descriptive statistics (mean and standard error) of FW, MDT of different BEN states are displayed in Figures 4A,B. We can see that FW of State 1 was relatively lower than other states, while the MDT of State 1 and State 4 were much higher than that of State 2 and State 3. The state transition probability matrix is presented in Figure 4C, from which we can find that: (a) no state transition occurred between two extreme low/high BEN states (State 1 and State 4); (b) transition probabilities from extreme states to intermediate states were higher than the opposite transition probabilities (see Supplementary Figure S3 for detailed information); (c) the largest between-state transition probabilities occurred between two intermediate states (State 2 and State 3; see Supplementary Figure S3). The stationary probability (Figure 4D), representing the expected behavior of the system in the long run, was further approximated from the state transition probability matrix (Allen et al., 2014). We can find that the stationary probabilities of two intermediate states were higher than those of two extreme states, demonstrated by the non-overlapped confidence interval between these two kinds of states.

**Figure 5:**
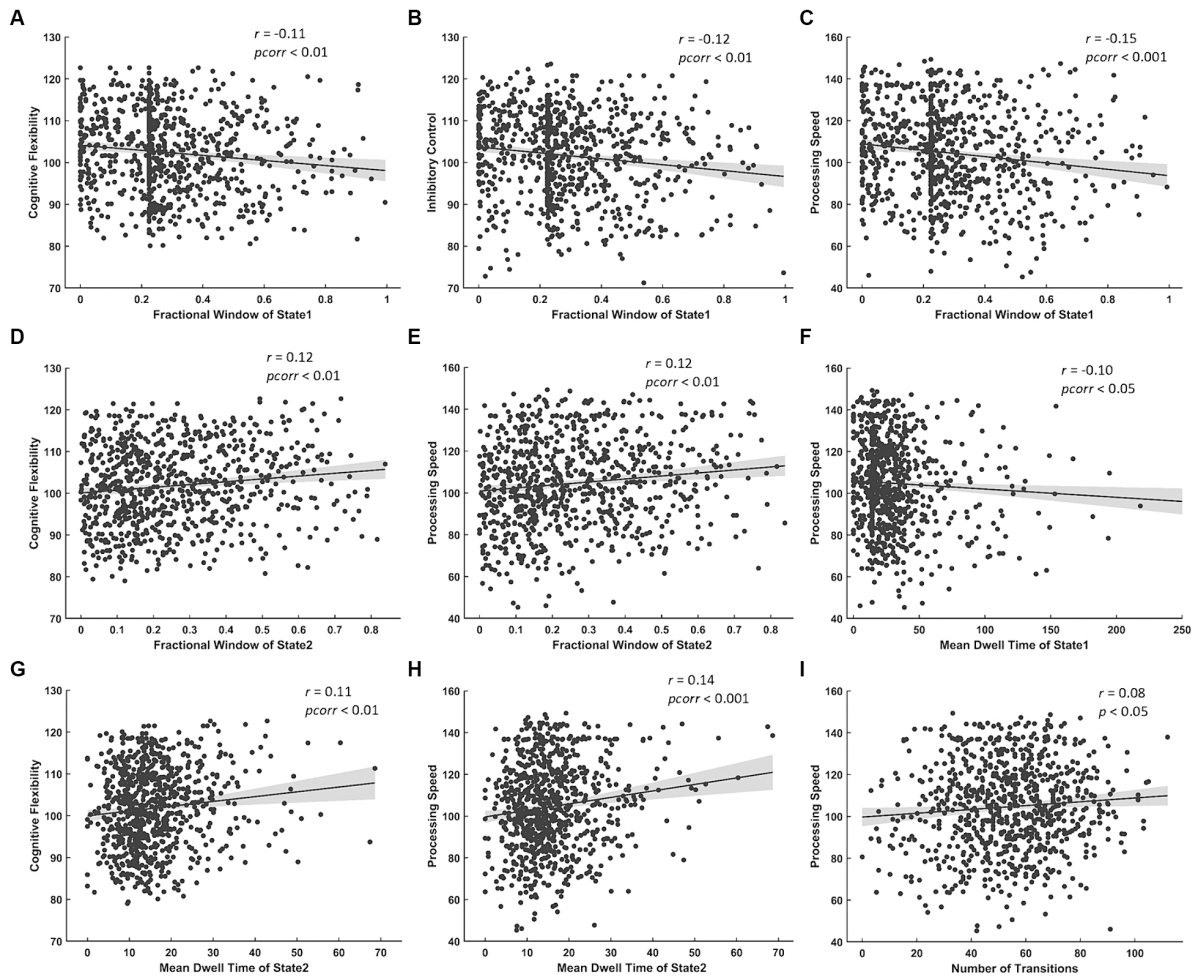
Significant correlations between temporal properties of BEN state and cognitive test scores. Negative correlations between FW of State 1 and **(A)** cognitive flexibility, **(B)** inhibitory control, **(C)** processing speed. Positive correlations between FW of State 2 and **(D)** cognitive flexibility, **(E)** processing speed. Negative correlation between MDT of State 1 and **(F)** processing speed. Positive correlations between MDT of State 2 and **(G)** cognitive flexibility, **(H)** processing speed. Positive correlation between NT and **(I)** processing speed.

The results of correlation analysis between temporal properties of BEN states and cognitive test scores show: (a) FW of State 1 was negatively correlated with cognitive flexibility, inhibitory control and processing speed (Figures 5A–C) while FW of State 2 was positively correlated with cognitive flexibility and processing speed (Figures 5D,E); (b) MDT of State 1 was negatively correlated with processing speed (Figure 5F) while that of State 2 was positively correlated with cognitive flexibility and processing speed (Figures 5G,H); (c) NT was positively correlated with processing speed (Figure 5I).

## 4 Discussion

The present study, to our knowledge, is the first to explore the time-varying features of BEN. The dBEN was derived by a sliding window approach. Based on this we identified four reoccurring whole-brain BEN states and their associated dynamics using k-means clustering analysis. The BEN states were distinguished from each other in terms of the overall BEN as well as BEN pattern across the whole-brain functional networks. The overall BEN values of different states were significantly different from each other except between two intermediate states, which, however, exhibited the lowest whole-brain BEN pattern similarity. From the perspective of temporal properties of BEN states, we find MDT of intermediate states was much lower than that of the extreme states, which is in line with the finding that state transitions occurred most frequently between two intermediate states. Such a finding indicates that these states are more active than the extreme states. We also find that the extreme states were more likely to transform into intermediate states, which is in line with that intermediate states exhibited higher stationary probabilities than the extreme states. It suggests that in the long run, the system is more likely to be found in State 2 or State 3. Since intermediate BEN is considered to correspond to higher neural complexity than extremely low/high BEN (Yang and Tsai, 2013; Yang et al., 2015; Xin et al., 2021), these findings implicate a healthy brain’s preference for higher whole-brain neural complexity, which supports the statement that sufficient neural complexity is essential for a healthy physiologic system (Goldberger et al., 2002a,b).

Importantly, our study identified significant correlations between temporal properties of BEN states and general cognitive ability. More specifically, the FW and MDT of State 1 were negatively correlated with cognitive flexibility and processing speed while the same temporal properties of State 2 were positively correlated with these cognitive test scores. The NT between different states was positively correlated with processing speed. These suggest that whole-brain BEN dynamics can also serve as an indicator of cognitive ability that is different from those correlated with the “”static” BEN. The negative association between temporal properties of State 1 and cognitive ability can be explained by the low whole-brain neural complexity corresponding to State 1, as reduced neural complexity indicates the degradation of the ability to adapt to the ever-changing environments and the consequential decrease of general cognitive function (Goldberger et al., 2002a,b; Hager et al., 2017). The positive correlation between temporal properties of State 2 and cognitive ability is not only in line with its corresponding high whole-brain neural complexity, but also related to the notion called metastable brain state. Metastable brain state refers to the flexible brain configuration that resides in the middle of a continuum situated between extreme chaos and extreme rigidity. Such a brain pattern is suggested necessary for the brain’s cognitive, behavioral, and social functions (Tognoli and Kelso, 2014; Kringelbach et al., 2015; Nomi et al., 2017). In the present study, two extreme BEN states correspond to extreme chaos and extreme regularity while State 2 corresponds to the metastable brain state. ^5^ Therefore, the relation between metastable brain state and cognitive functions is supported. The positive correlation between NT and processing speed is in line with the above-mentioned correlations between the temporal properties of State 1/State 2 and processing speed: since state transitions occurred less frequently in State 1 and more frequently in State 2, higher processing speed which is associated with lower occurrence of State 1 and higher occurrence of State 1. Such a finding is also consistent with previous dFC research which has revealed that higher NT indicates higher flexibility of the system, and is linked with better cognitive performances (Li et al., 2017; Fiorenzato et al., 2019).

It is worth noting that although the two intermediate BEN states all exhibit similar whole-brain neural complexity and have the characteristics of metastable brain state, a significant positive correlation with cognitive ability only existed in the temporal properties of State 2. Since State 2 and State 3 have the lowest BEN pattern similarity, it is reasonable to speculate that BEN pattern also contributes to the underlying cognitive function of BEN state. In our case, BEN pattern dissimilarity between these two states mainly lies in the locations of ICs with low within-state BEN: those in State 2 were located in DMN, ECN, and part of SAN, while those in State 3 were located in SMN (except CBN) and VN. The brain regions of within-state low BEN in State 2, largely overlap with the highlighted brain areas (DMN and ECN) reported in Wang (2021) and Lin et al. (2022) where BEN was a negative predictor for general brain functionality, which is solid support of our speculation. Taken together, our findings under the “dynamic” perspective reconcile the controversial relation between “static” BEN and general cognitive ability mentioned in Section 1 (Wang, 2021): although the whole-brain BEN configuration with a low overall value seems to be harmful to general cognitive ability, the whole-brain BEN configuration, with overall intermediate value and within-state low BEN located in DMN, ECN and part of SAN, facilitates general cognitive ability. As a result, it leads to a negative association between BEN and general cognitive ability in these brain regions as reported by Wang (2021).

There are several limitations of the present study. Firstly, our study adopted a conservative window length (100 TR) to derive dBEN, as a window length of around 20 TR is sufficient for dFC analysis (Preti et al., 2017). Due to the long time points of the HCP dataset, such a setting still can generate a sufficient number of sliding windows. However, since many fMRI datasets are of much shorter time points (100–200), such a setting may lead to an insufficient number of sliding windows. Future research is needed to optimize this parameter for the application of dBEN analysis on the fMRI dataset with short time points. Secondly, individuals with mental or neuropsychiatric disorders are often accompanied by disrupted cognitive functions. For example, patients with attention-deficit/hyperactivity disorder, obsessive-compulsive disorder, and autism are associated with impaired cognitive flexibility (Barkley, 2015; Rosa-Alcázar et al., 2020; Lage et al., 2023). Since our study revealed cognitive flexibility is highly associated with temporal properties of BEN state, it is reasonable to assume abnormal BEN dynamics in participants with such disorders. Future research may adopt a similar analysis framework to investigate altered temporal properties of whole-brain BEN states in these clinical populations and their associations with certain symptom severity. Thirdly, it has been suggested that the brain is a dynamic system that the evolution of local activities entangles with the reconfiguration of brain interactions (Gross and Blasius, 2008; Fu et al., 2021), it would be meaningful to explore the covarying relations between dBEN and dFC (even dynamical network topology) in the future as such investigation may provide novel information about the neural mechanism of cognitive function that cannot be obtained by investigating either of them alone. Fourthly, recent studies applied fuzzy entropy to the dynamic resting-state functional network to evaluate the temporal variability of the dynamic resting-state brain networks (Li et al., 2021; Jiang et al., 2022). They found such temporal variability was related to cognitive processes and could serve as a potential biomarker for mental disorders. These results provide a second-stage analytical direction for dBEN in the future.

## 5 Conclusion

Here, we presented the first attempt to identify reoccurring patterns in BEN in the resting state. Out of the four BEN states we identified, two were correlated with general cognitive abilities. The FW and MDT of one State, characterized by the extremely low overall BEN, were negatively correlated with cognitive flexibility and processing speed. The FW and MDT of another State, characterized by its overall intermediate value and within-state low BEN located in DMN, ECN, and part of SAN, were positively correlated with those cognitive abilities. These four whole-brain BEN states differ from each other in terms of characteristics of centroidal BEN (including overall BEN value and BEN pattern distribution across whole-brain functional networks), which leads to differences in their temporal properties and their associations with general cognitive ability. The present study advances our understanding of the BEN dynamics by providing the first piece of evidence of reoccurring BEN patterns and their associations with cognitive ability. It can serve as a framework for future investigations exploring abnormal BEN dynamics in clinical populations.

## Data availability statement

The original contributions presented in the study are included in the article/Supplementary material, further inquiries can be directed to the corresponding author.

## Ethics statement

Ethics approval was granted by the Ethics Committee of the Center for Psychological Sciences, Zhejiang University. The study was conducted in accordance with the local legislation and institutional requirements. Written informed consent for participation was not required from the participants or the participants’ legal guardians/next of kin in accordance with the national legislation and institutional requirements.

## Author contributions

XX: Conceptualization, Data curation, Formal analysis, Investigation, Methodology, Software, Visualization, Writing – original draft. JY: Data curation, Formal analysis, Investigation, Software, Validation, Visualization, Writing – original draft, Writing – review & editing. XG: Conceptualization, Funding acquisition, Project administration, Resources, Supervision, Validation, Visualization, Writing – review & editing.

## Footnotes

^1^It should be noted that Wang (2021) calculated the temporal stability of BEN using a sliding window approach, without exploring the reoccurring BEN patterns.

^2^https://db.humanconnectome.org/data/projects/HCP_1200

^3^findlab.stanford.edu/functional_ROIs.html

^4^https://wiki.humanconnectome.org/display/PublicData/HCP-YA+Data+Dictionary-+Updated+for+the+1200+Subject+Release

^5^As BEN is a negative predictor of the regularity of brain activity, State 1 and State 4 which are of the extreme low and extreme high whole-brain BEN among all states, are naturally corresponded to extreme regular and extreme irregular (chaos) whole-brain activity.
