# Supplementary material for: The brain entropy dynamics in resting state
Source: Front Neurosci. 2024 Mar 26;18:1352409. doi: 10.3389/fnins.2024.1352409 (PMC11002175; doi:10.3389/fnins.2024.1352409)
Supplement: Supplementary file 1 [file Data_Sheet_1.docx]

Supplementary Material

# Selection of BEN parameters (*m* and *r*)

The selection of pattern length (*m*) and tolerance factor (*r*) was based on the strategy of minimization relative error of BEN in CSFs (Yang et al., 2018), which is based on the viewpoint that CSF signals exhibited the characteristics of uncorrelated noise (Wu et al., 2012). For the present study, we adopted a grid search method to calculate the relative error of dBEN in CSF in a range of *m* (1, 2, 3) and *r* (0.25 to 0.7 in the step of 0.05). The settings of the range is according to previous BEN studies (e.g., Sokunbi, 2014; Wang, 2021; Yang et al., 2018). From Figure 1S, we can find *m* = 2, *r* = 0.55 are the parameters corresponding to minimal relative error of dBEN in CSF.


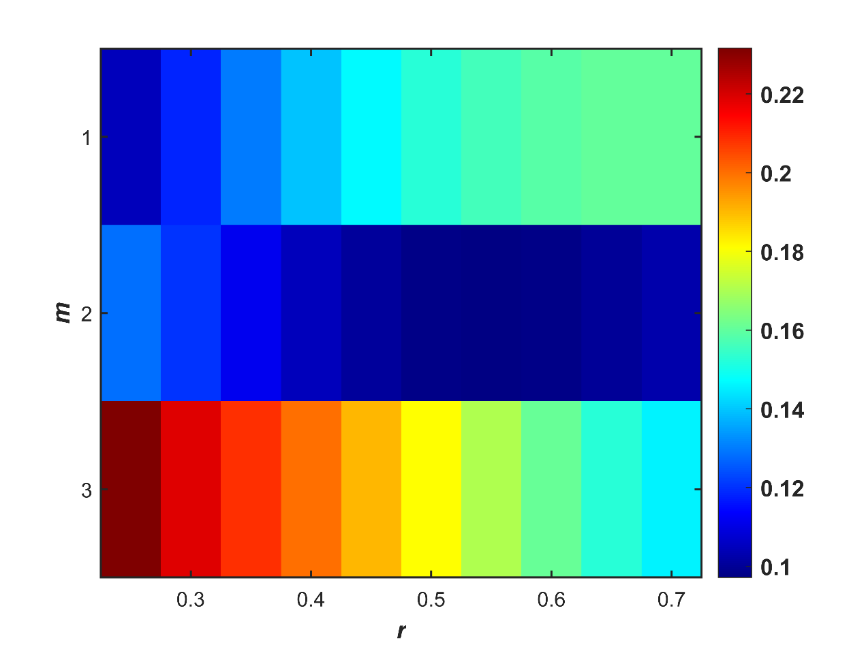


**Figure S1.** Color map of the relative error of dBEN of CSF in a range of of *m* and *r*.

# Cluster number validity analysis

To reduce redundancy between windows as well as computational demand, windows consisting of local maxima variance in dBEN matrix were used as subsample (a total of 135231 windows, 166.53 ± 9.53 windows per subject) (Kim et al., 2017). We performed a cluster number validity analysis (using elbow and silhouette criteria) on the subsample to find the optimal cluster number (*k*, ranging from 2 to 10). The optimal cluster number (*k*) was determined to be 4 (Figure S2).


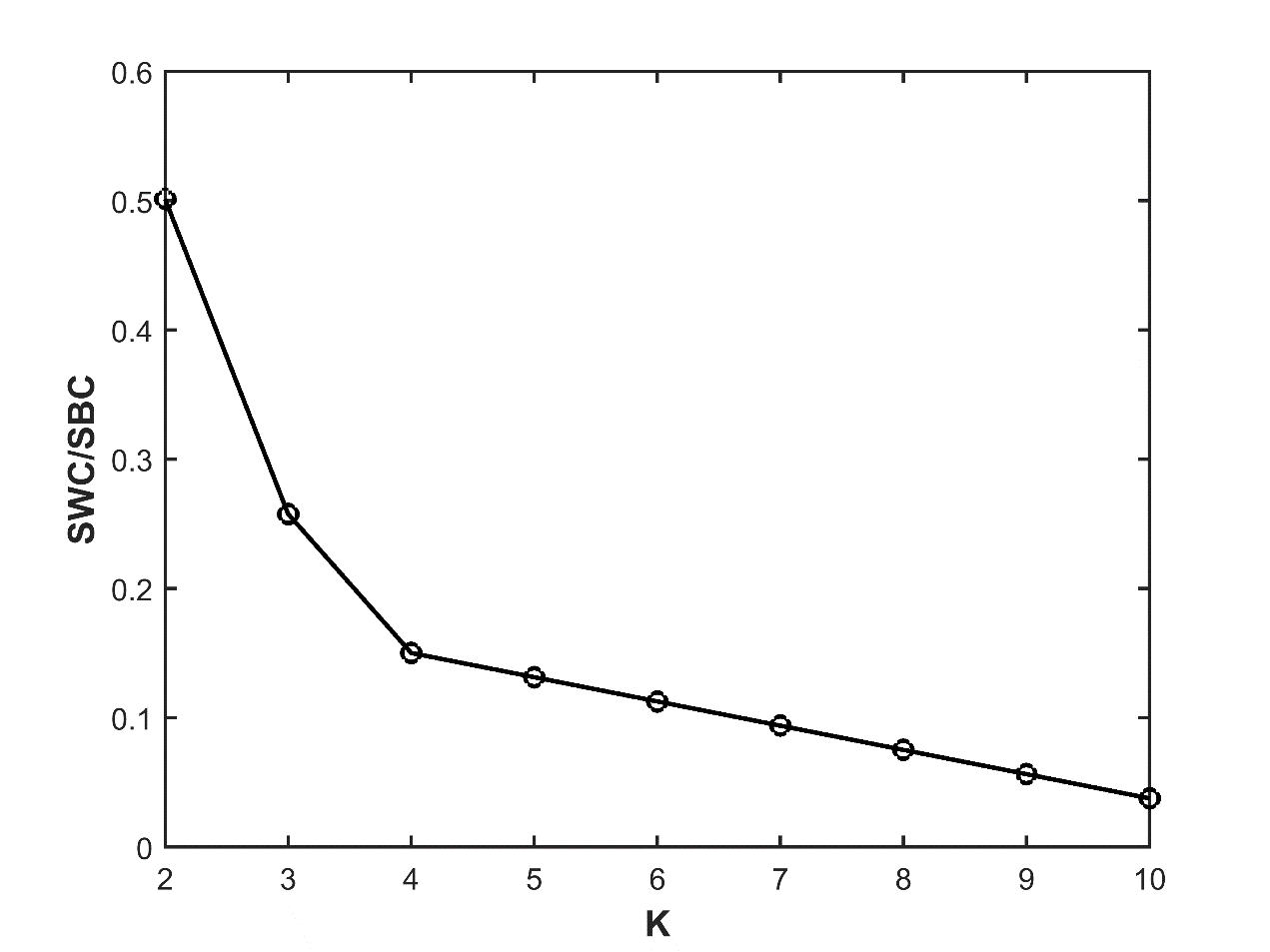


**Figure S2.** The visualized results of cluster number validity analysis using elbow criteria. SWC and SBC represent the sum of within cluster distance and the sum of between cluster distance respectively.


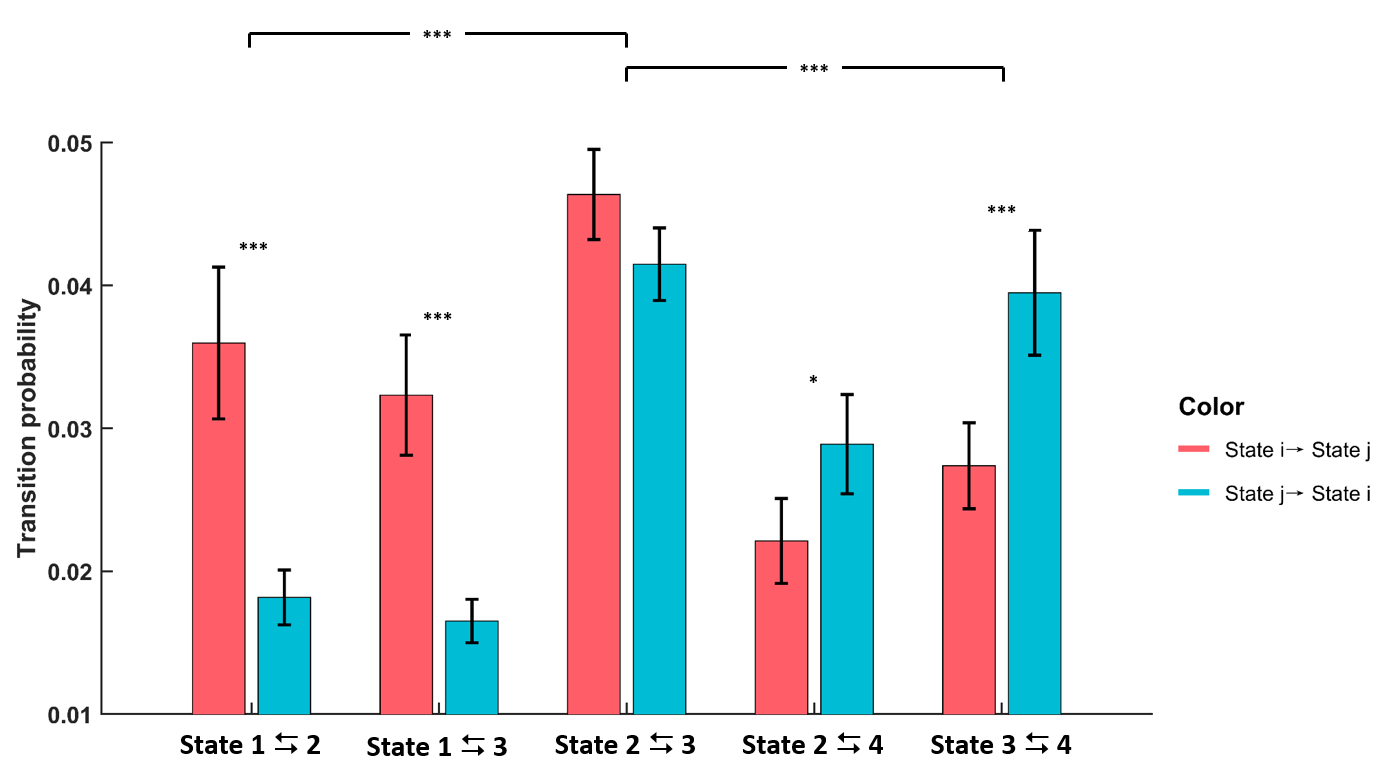


**Figure S3.** Comparison of different state transition probabilities, where the hot column represents the transition probability from BEN state with small index (State *i*) to the state with large index (State *j*), and the cool column represents the transition probability with opposite direction. Note ^***^ and ^*^ represent *pcorr* < 0.001 and *pcorr* < 0.05 respectively (paired-sample *t* test, FDR corrected).

**Table S1.** Detailed information of the meaningful ICs

| IC Index | Brain area (AAL atlas) | peak MNI coordinates of spatial maps | | | *r^[[1]](#footnote-1)^* |
| --- | --- | --- | --- | --- | --- |
|  |  | x | y | z |  |
| 1. IC 50 | Thalamus_B | 12 | -13 | 10 | 0.43 |
| 1. IC 60 | Putamen_R | 24 | 10 | -2 | 0.15 |
|  | Putamen_L | -24 | 10 | -7 |  |
| 1. IC 61 | Caudate_R | 10 | 0 | 14 | 0.27 |
|  | Caudate_L | -7 | 2 | 10 |  |
| 1. IC 80 | Thalamus_B | 3 | -3 | 2 | 0.24 |
| 1. IC 91 | Caudate_L | -14 | 16 | 6 | 0.16 |
| 1. IC 20 | Temporal_Mid_L | -47 | -38 | 0 | 0.25 |
| 1. IC 31 | Temporal_Mid_R/SupraMarginal_R | 64 | -46 | 26 | 0.20 |
| 1. IC 43 | Temporal_Sup_R | 66 | -20 | 13 | 0.39 |
|  | Temporal_Sup_L | -64 | -20 | 12 |  |
| 1. IC 6 | Occipital_Mid_L | -34 | -82 | 40 | 0.53 |
|  | Occipital_Mid_R/Angular_R | 43 | -76 | 36 |  |
| 1. IC 13 | Precuneus_B/Cuneus_B | 10 | -76 | 40 | 0.73 |
| 1. IC 14 | Occipital_Mid_R/Parietal_Sup_R/  Precuneus_R | 42 | -80 | 32 | 0.33 |
|  | Precuneus_L | -12 | -74 | 52 |  |
| 1. IC 21 | Frontal_Sup_Medial_B/ Frontal_Sup_B | -13 | 57 | 30 | 0.32 |
| 1. IC 22 | Frontal_Sup_Medial_B | 2 | 60 | 16 | 0.65 |
| 1. IC 27 | Temporal_Mid_R/Angular_R | 60 | -57 | 20 | 0.22 |
|  | Temporal_Mid_L/Angular_L | -53 | -57 | 20 |  |
|  | Precuneus_B | 0 | -62 | 43 |  |
| 1. IC 3 | Angular_R/Parietal_Inf_R | 54 | -64 | 36 | 0.27 |
| 1. IC 7 | Parietal_Inf_R/SupraMarginal_R | 57 | -36 | 50 | 0.31 |
| 1. IC 8 | Angular_L/Parietal_Inf_L | -46 | -67 | 44 | 0.29 |
| 1. IC 16 | Temporal_Inf_L/Temporal_Mid_L | -56 | -46 | -12 | 0.26 |
|  | Temporal_Mid_R/Temporal_Inf_R | 62 | -43 | -10 |  |
|  | Parietal_Sup_L/Parietal_Inf_L | -26 | -74 | 47 |  |
|  | Angular_R/Parietal_Sup_R | 38 | -68 | 501 |  |
| 1. IC 17 | Frontal_Mid_L/Frontal_Inf_Tri_L | -47 | 34 | 24 | 0.29 |
|  | Parietal_Inf_L | -52 | -47 | 50 |  |
| 1. IC 32 | Frontal_Mid_L/Frontal_Mid_Orb_L | -43 | 52 | 2 | 0.27 |
|  | Frontal_Mid_R/Frontal_Mid_Orb_R | 42 | 56 | 0 |  |
| 1. IC 49 | Cerebelum_Crus2_R | 30 | -72 | -47 | 0.26 |
|  | Cerebelum_Crus2_L | -30 | -72 | -47 |  |
| 1. IC 59 | Cerebelum_Crus2_L/Cerebelum_Crus1_L | -32 | -72 | -38 | 0.25 |
|  | Cerebelum_Crus2_R/Cerebelum_Crus1_R | 34 | -70 | -40 |  |
| 1. IC 9 | Parietal_Inf_L/Angular_L | -57 | -52 | 40 | 0.27 |
|  | Parietal_Inf_R/SupraMarginal_R | 57 | -50 | 44 |  |
| 1. IC 11 | Parietal_Inf_L/SupraMarginal_L | -60 | -36 | 44 | 0.51 |
|  | SupraMarginal_R | 62 | -38 | 42 |  |
| 1. IC 25 | Postcentral_R/SupraMarginal_R | 57 | -22 | 50 | 0.19 |
|  | Temporal_Inf_R | 54 | -60 | -10 |  |
| 1. IC 26 | SupraMarginal_L/Temporal_Sup_L | -60 | -30 | 18 | 0.31 |
|  | SupraMarginal_R | 50 | -30 | 28 |  |
|  | Parietal_Sup_L/Precuneus_L | -18 | -50 | 64 |  |
|  | Postcentral_R/Parietal_Sup_R | 20 | -50 | 70 |  |
| 1. IC 28 | Frontal_Mid_L | -30 | 52 | 26 | 0.43 |
|  | Frontal_Mid_R | 32 | 52 | 28 |  |
| 1. IC 39 | Frontal_Inf_Tri_L/Frontal_Inf_Oper_L/Frontal_Inf_Orb_L | -47 | 20 | -6 | 0.23 |
|  | Precentral_L | -50 | 2 | 50 |  |
|  | Supp_Motor_Area_L | -2 | 6 | 66 |  |
| 1. IC 24 | Postcentral_L | -53 | -7 | 24 | 0.15 |
|  | Postcentral_R | 53 | -6 | 24 |  |
| 1. IC 34 | Postcentral_L/ Parietal_Inf_L | -62 | -18 | 30 | 0.12 |
|  | Postcentral_R | 57 | -13 | 30 |  |
|  | Precentral_L | -60 | 4 | 30 |  |
| 1. IC 35 | Parietal_Sup_R | 20 | -64 | 64 | 0.15 |
|  | Parietal_Sup_L | -20 | -62 | 64 |  |
| 1. IC 36 | Postcentral_L | -38 | -16 | 46 | 0.26 |
|  | Precentral_R | 50 | -7 | 54 |  |
|  | Supp_Motor_Area_B | 4 | 2 | 62 |  |
| 1. IC 41 | Postcentral_R/Precentral_R | 42 | -22 | 64 | 0.32 |
| 1. IC 42 | Postcentral_L/Precentral_L | -37 | -24 | 66 | 0.30 |
| 1. IC 97 | Vermis_4_5/Cerebelum_4_5_B | 8 | -52 | -8 | 0.29 |
| 1. IC 98 | Cerebelum_4_5_R/Cerebelum_6_R | 16 | -56 | -16 | 0.22 |
|  | Cerebelum_4_5_L/Cerebelum_6_L | -18 | -56 | -16 |  |
| 1. IC 1 | Occipital_Mid_L/ Occipital_Inf_L | -26 | -100 | -6 | 0.27 |
|  | Occipital_Inf_R/Lingual_R/Calcarine_R | 28 | -98 | -6 |  |
| 1. IC 2 | Occipital_Mid_L/Occipital_Inf_L | -36 | -92 | 3 | 0.56 |
|  | Occipital_Inf_R/Occipital_Mid_R | 40 | -87 | -2 |  |
| 1. IC 12 | Calcarine_B | 10 | -72 | 13 | 0.57 |
| 1. IC 23 | Occipital_Mid_L | -24 | -96 | 2 | 0.26 |
| 1. IC 29 | Calcarine_B | -7 | -93 | -2 | 0.30 |
| 1. IC 33 | Occipital_Mid_R/Calcarine_R | 26 | -98 | 10 | 0.41 |
| 1. IC 37 | Lingual_L/Calcarine_L | -10 | -94 | -12 | 0.26 |
|  | Lingual_R | 14 | -92 | -6 |  |

Abbreviations: IC, independent component; L, left; R, right; Ant, anterior; Inf, inferior; Med, medial; Mid, middle; Sup, superior; Supp, supplementary; Oper, opercularis; Orb, orbital; Tri, triangularis.

# References

Kim, J., Criaud, M., Cho, S. S., Díez-Cirarda, M., Mihaescu, A., Coakeley, S., ... & Strafella, A. P. (2017). Abnormal intrinsic brain functional network dynamics in Parkinson’s disease. *Brain*, *140*(11), 2955-2967.

Sokunbi, M. O. (2014). Sample entropy reveals high discriminative power between young and elderly adults in short fMRI data sets. *Frontiers in neuroinformatics*, *8*, 69.

Wang, Z. (2021). The neurocognitive correlates of brain entropy estimated by resting state fMRI. *Neuroimage*, *232*, 117893.

Wu, C. W., Gu, H., Zou, Q., Lu, H., Stein, E. A., & Yang, Y. (2012). TE-dependent spatial and spectral specificity of functional connectivity. *Neuroimage*, *59*(4), 3075-3084.

Yang, A. C., Tsai, S. J., Lin, C. P., & Peng, C. K. (2018). A strategy to reduce bias of entropy estimates in resting-state fMRI signals. *Frontiers in neuroscience*, *12*, 398.

1. The parameter *r* refers to the spatial correlation coefficient between each IC and a specific network of the Stanford functional ROI template ([findlab.stanford.edu/functional_ROIs.html](http://findlab.stanford.edu/functional_ROIs.html)). [↑](#footnote-ref-1)
